# Circadian Rhythms, the Mesolimbic Dopaminergic Circuit, and Drug Addiction

**DOI:** 10.1100/tsw.2007.213

**Published:** 2007-11-02

**Authors:** Colleen A. McClung

**Affiliations:** Department of Psychiatry and Center for Basic Neuroscience, The University of Texas, Southwestern Medical Center, 5323 Harry Hines Blvd., Dallas, TX 75390-9070, USA

**Keywords:** circadian, drug abuse, dopamine, clock, striatum, ventral tegmental area, nucleus accumbens

## Abstract

Drug addiction is a devastating disease that affects millions of individuals worldwide. Through better understanding of the genetic variations that create a vulnerability for addiction and the molecular mechanisms that underlie the progression of addiction, better treatment options can be created for those that suffer from this condition. Recent studies point to a link between abnormal or disrupted circadian rhythms and the development of addiction. In addition, studies suggest a role for specific genes that make up the molecular clock in the regulation of drug sensitivity, sensitization, and reward. The influence of circadian genes and rhythms on drug-induced behaviors may be mediated through the mesolimbic dopaminergic system. This system has long been implicated in the development of addiction, and recent evidence supports a regulatory role for the brain's central pacemaker and circadian gene expression in the regulation of dopaminergic transmission. This review highlights the association between circadian genes and drug addiction, and the possible role of the mesolimbic dopaminergic system in this association.

## INTRODUCTION

Drug addiction has long been associated with major disruptions in the sleep/wake cycle, as well as abnormal rhythms in body temperature, hormone levels, and blood pressure[1,2]. Some of these disruptions are caused by chronic exposure to the stimulant or depressant properties of the drugs themselves. Indeed, chronic exposure to alcohol and other drugs lead to fundamental changes in the circadian free-running period in rodents, and significant changes in the sleep/wake cycle in humans[3]. Withdrawal from these drugs can also have persistent long-lasting effects on these daily rhythms in recovering addicts. These disruptions are highly problematic and often lead to relapse[2,4]. Addiction may also be more prevalent in individuals that have a compromised or nonfunctional molecular clock. Addictive disorders are very often comorbid with mood disorders or other psychiatric conditions, and many of these disorders are thought to be related to a disrupted circadian clock[5,6,7,8,9,10]. Recent studies also find that individuals with a variety of sleep problems are more susceptible to substance abuse[11]. Drugs of abuse can also have a seasonal pattern, with an increase in the use of alcohol and other drugs typically in the winter, when certain individuals are more susceptible for depression[12,13]. The circadian clock also regulates drug responsiveness, since there is a diurnal variation in the sensitivity to nearly all types of illicit drugs in the human population. In fact, retrospective studies that examine the admission of drug overdose victims to urban hospitals find the greatest presentation around 6:30 p.m. vs. other times of day indicating a diurnal effect[14]. Taken together, these results suggest that the physical response to these drugs and the vulnerability for addiction are both influenced by the circadian clock.

## THE MASTER AND PERIPHERAL CLOCKS

Circadian rhythms are largely controlled by a transcriptional feedback loop located in the suprachiasmatic nucleus (SCN). The SCN sends signals throughout the brain and to peripheral "clocks" in other organs in the body. These clocks regulate the rhythms in many bodily functions including sleep/wake activity, hormone levels, body temperature, and digestive processes[15,16]. There are also circadian rhythms in higher brain functions, such as cognition and attention, as well as daily and seasonal rhythms in mood[10,17,18,19,20]. One of the major outputs of the circadian clock is to the pineal gland, resulting in the rhythmic release of the hormone melatonin. Melatonin (often called the "hormone of darkness") is released in the evening and its levels are suppressed by light[21]. Melatonin rhythms are also seasonal in many organisms, with greater levels produced and released in the winter, and it is involved in seasonal reproduction and hibernation[21]. Melatonin in humans acts as a sleep facilitator and disruptions in normal melatonin rhythms have been linked to jet lag, shift-work sleep disorder, and seasonal affective disorder[22,23,24]. The receptors for melatonin are expressed widely throughout the brain, including expression in many limbic regions and in the SCN where melatonin can feed back on the molecular clock[25].

The molecular clock in all organisms is essentially a series of interconnected transcriptional loops that are regulated over the course of 24 h. In mammals, the circadian locomotor output cycles kaput (CLOCK) and brain and muscle Arnt-like protein-1 (BMAL1) proteins dimerize and induce expression of the *Period* genes (*Per1*, *Per2*, and *Per3*), the *Cryptochromes* (*Cry1* and *Cry2*), and many other genes (Fig. 1)[16]. After a time lag, the PER and CRY proteins enter the nucleus, and CRY proteins inhibit the actions of CLOCK:BMAL1. Casein kinase 1 (CK1) ε and δ, and glycogen synthase kinase 3β (GSK3β), phosphorylate these circadian proteins, leading to changes in protein stability, dimerization, and nuclear entry[26]. Protein phosphatase 5 (PP5) is also involved in regulating the post-translational modifications necessary for circadian function[27]. The orphan nuclear receptor REV-ERBα and the transcriptional regulator RORA also participate in adjoining feedback loops, where they regulate the expression of *Bmal1*[16]. In forebrain regions, neuronal PAS domain protein 2 (NPAS2), a protein very similar in sequence and function to CLOCK, can regulate the expression of the *Per* and *Cry* genes[28]. NPAS2 has particularly high expression in striatal regions of the brain, and has been implicated in the formation of emotional memory, sleep, and food entrainment[29,30,31]. NPAS2 may also substitute for CLOCK in the SCN under conditions in which CLOCK function has been disrupted[32].

Though the master circadian pacemaker is located in the SCN, these proteins are expressed throughout the brain, and there are many indications that they form SCN-independent pacemakers in other brain regions that control the entrainment to food and other nonphotic stimuli[33,34]. These additional clocks can desynchronize from the SCN in response to certain types of stimuli. For example, the circadian activity rhythms in rodents can be entrained to daytime methamphetamine injections, and methamphetamine in the drinking water can induce a robust activity rhythm in SCN-lesioned (thus otherwise arrhythmic) animals[33,35]. Interestingly, treatment with methamphetamine shifts the expression of the *Per* genes in striatal regions of the brain in a manner that matches the shift in activity rhythms[33]. Molecular rhythms in the SCN and in melatonin levels remain unaffected by these treatments. Thus, there is a disconnect between the SCN, molecular rhythms in the striatum, and locomotor activity rhythms with administration of psychoactive drugs, suggesting that these non-SCN

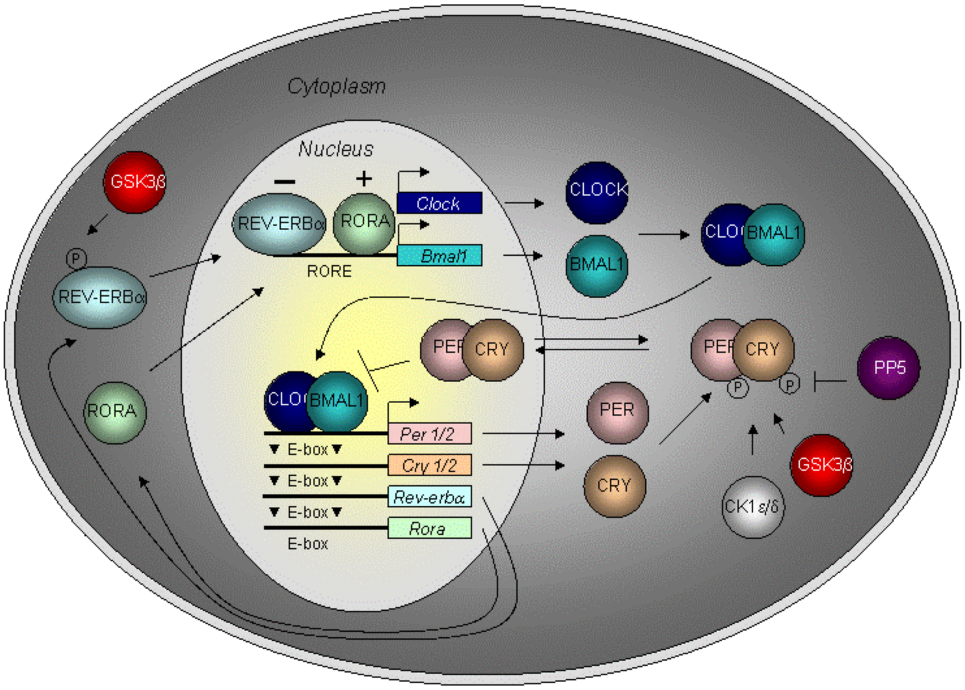

**FIGURE 1.** A simplified model of the molecular clock. CLOCK and BMAL1 induce the expression of the *Per*, *Cry*, *Rev-erbα*, and *Rora* genes (among many others). In certain regions of the brain, NPAS2 can substitute for CLOCK and affect transcription of these genes. PER and CRY inhibit the actions of CLOCK:BMAL1. RORA can positively influence *Bmal1* transcription, while REV-ERBα negatively regulates *Bmal1*. CK1ε and δ, and GSK3β, phosphorylate several proteins in these feedback loops. PP5 can dephosphorylate these proteins. Additional circadian factors and means of regulation are continuously being identified, thus this represents a working model.

clocks may be involved in drug responsiveness. Furthermore, it is possible that the long-term desynchronization of the molecular clock in striatal regions from the SCN could lead to alterations in mood, motivation, or other processes associated with addiction.

## DIURNAL DIFFERENCES IN DRUG-INDUCED BEHAVIORAL RESPONSES

Studies in animal models of addiction have found a diurnal difference in drug sensitivity, sensitization, conditioned preference, and self-administration. Rats have an increase in the sensitivity to the reinforcing properties of cocaine at 1:00 a.m. and 1:00 p.m. vs. rats tested at 7:00 a.m. or 7:00 p.m., as indicated by drug self-administration at lower doses and a decrease in the overall drug intake at these time periods[36]. This difference in sensitivity is not due to changes in the pharmacokinetics of cocaine since they are similar at all times of day[36]. The development of cocaine sensitization also differs depending on the time of day in which the drug is given. In CBA/J mice, sensitization is greater when cocaine is given during the day than during the night[37]. This also correlates with an increase in *Per1* levels in striatal regions during the day vs. the night in these mice[37,38]. Interestingly, mice in the AKR/J strain, which are deficient in melatonin, do not have this diurnal variation in sensitization or *Per1* striatal expression, suggesting that these differences might be mediated by melatonin[37]. Indeed, pinealectomy also abolishes *Per1* rhythms in striatal regions, but has no effect on rhythms in other limbic regions of the brain, including the oval nucleus of the bed nucleus of the stria terminalis, the central nucleus of the amygdala, and the hippocampus[39]. Thus, the influence of melatonin on *Per* gene expression in striatal regions may be somewhat unique. Intriguingly, other studies using a rat model have found that, in contrast to short-term sensitization, long-term cocaine sensitization (observed 2 weeks after the last injection) is greater when the drug is given at the onset of the dark phase (ZT12)[40]. A single treatment with melatonin 15 min before the last cocaine injection at an earlier timepoint (ZT6) enhanced this long-term sensitization 2 weeks later, suggesting that melatonin is involved in the long-term sensitization to cocaine. In addition to drug self-administration and locomotor sensitization, studies have found that conditioned place preference for cocaine also has a diurnal rhythm, and the effects are greater during the day (ZT5) vs. the night (ZT20)[41]. This difference is also dependent on melatonin, since pinealectomy abolishes this rhythm in response[41].

## DIURNAL REGULATION OF DOPAMINERGIC TRANSMISSION

Melatonin could influence cocaine sensitization and preference through modulation of the diurnal rhythms in dopamine transmission. Indeed, rhythms in cocaine sensitivity correlate with diurnal alterations in postsynaptic levels of dopamine and the activity of dopaminergic receptors in striatal regions[42,43]. The midbrain dopaminergic projection from the ventral tegmental area (VTA) to the nucleus accumbens (NAc) is known to play an important role in drug addiction. Furthermore, virtually all elements of dopaminergic transmission have a diurnal rhythm, including the expression of the dopamine receptors, the dopamine transporter (DAT), and the rate limiting enzyme in dopamine synthesis, tyrosine hydroxylase (TH)[44,45,46,47,48,49,50,51]. A recent study by Sleipness and colleagues found that the diurnal rhythms in expression of DAT and TH in the NAc are dampened in SCN-lesioned animals vs. sham controls, suggesting that the SCN is important in maintaining proper rhythmicity of these genes[50]. Pinealectomy also blunts the diurnal rhythms of dopamine in the striatum and these rhythms can be restored with administration of melatonin[52]. Melatonin receptors are expressed in both the VTA and NAc, and have a diurnal rhythm in expression in these areas with high levels during the day and low levels at night in the mouse[25]. Studies have shown that in the hypothalamus, retina, and hippocampus, melatonin has an inhibitory effect on dopamine release[53]. However, no inhibitory effect of melatonin was found in the striatum; therefore, it is still unclear as to how melatonin regulates the behavioral responses to drugs of abuse.

## ABNORMALITIES IN THE CIRCADIAN SYSTEM ASSOCIATE WITH CHANGES IN DRUG-INDUCED BEHAVIOR

In human populations, addiction appears to have a strong genetic component[54]. Furthermore, studies in animal models of addiction have found differences in free-running circadian rhythms between genetically inbred strains of rats and mice that display variations in drug preference. Rosenwasser and colleagues measured the free-running activity rhythms in animals that were selectively bred based on a high preference for ethanol or low preference for ethanol[55]. They found that the two ethanol-preferring lines (P, HAD) had a shorter free-running period than the low-preference lines (NP, LAD) when animals were housed in constant light. This difference also held true for the HAD line (but not the P line) when animals were housed in constant darkness. The HAD line also displayed a “splitting” of circadian activity in that they showed two distinct bouts of activity in constant light, which was not seen in the other lines. Similar shortening of the free-running period in constant darkness has also been found in ethanol-preferring mice compared to those selectively bred for low ethanol preference[56]. These results suggest that genetic ethanol preference is associated with abnormal circadian rhythms.

The original studies suggesting that individual circadian genes may play a key role in drug responsiveness were performed in *Drosophila* and showed that flies lacking a functional *Per*, *Clock*, *Cycle*, or *Doubletime* gene (but not the *Timeless* gene) all fail to sensitize to cocaine[57]. The fact that *Timeless* is not involved in this behavior suggests that perhaps these genes have independent functions in regulating cocaine sensitization that may not relate to their role in circadian rhythms. Indeed, a study by

Abarca et al. found that mice that are lacking a functional *mPer1* gene (*mPer1$^{Brdm1}$*) also fail to sensitize to cocaine or show a conditioned preference for cocaine, while mice with a mutant *mPer2* gene (*mPer2$^{Brdm1}$*) show an enhanced sensitization to cocaine, but normal levels of conditioned preference for cocaine[58]. Reduction in the expression of *mPer1* by DNAzyme targeting also leads to a reduction in the conditioned preference for morphine, suggesting that mPER1 may affect the rewarding properties of multiple substances[59,60]. However, further studies with the *Per* gene knock-out mice found that the *mPer2* mutant mice show a greater level of self-administration and incentive for alcohol than wild-type mice, while the response of the *mPer1* mutant mice in these paradigms is not different from wild-type mice[61,62]. Therefore, these proteins may perform separate functions in the regulation of the reward value for specific drugs of abuse. In support of this hypothesis, a study by Yuferov et al. found that *rPer1* was induced in the caudate putamen (CPu) following acute cocaine, while *rPer2* was only induced following a chronic “binge” pattern of cocaine[63]. Furthermore, acute methamphetamine treatment leads to a rapid induction of *mPer1*, but not *mPer2* or *mPer3* expression in the CPu[64]. There also appears to be regional differences in the induction of these genes within the striatum between the NAc and CPu in that there is selective induction of the *Per* genes in each of these regions in response to psychostimulants. This is interesting since the NAc is more traditionally associated with drug reward, while the CPu is more involved in the changes in activity levels with drug administration, as well as the habitual and compulsive properties of drug seeking and taking[65]. Therefore, these proteins appear to play selective roles in these striatal regions in the acute or chronic response to psychostimulants.

In addition to the *Per* genes, we have found that the *Clock* gene is important in setting the reward value for drugs like cocaine. Mice carrying a mutation (Δ19) in the *Clock* gene develop robust sensitization to cocaine, have an increase in cocaine preference, and an increase in the reward value for cocaine as measured by intracranial self-stimulation following cocaine treatment[66,67]. Interestingly, when we went on to test the *Clock* mutant mice in various measures of activity, hedonic state, depression, and anxiety, we found that the *Clock* mutant mice display a complete behavioral profile that is strikingly similar to the manic state of humans with bipolar disorder (Table 1)[67]. Bipolar disorder is highly comorbid with drug abuse disorders, and patients in the manic state are often drawn to psychostimulants, such as cocaine and amphetamine[68]. When we treat these mice with the mood stabilizer lithium, we are able to return many of their behavioral responses to wild-type levels[67]. Given the evidence linking the midbrain dopaminergic reward circuit to the regulation of mood and drug preference, we wanted to examine the levels of neuronal activity in this region in the *Clock* mutant mice[46,69]. We found that these mice have an increase in dopamine cell firing and bursting in the VTA[66]. This increase in dopaminergic activity was correlated with a number of gene expression changes in the VTA in these mice, including an up-regulation of TH levels and phosphorylation[66]. In addition, we are able to rescue their locomotor and anxiety-related behavioral responses when we expressed a functional CLOCK protein specifically in the VTA using viral-mediated gene transfer. This suggests that CLOCK likely functions in the VTA-NAc circuit to regulate dopaminergic synthesis, neuronal activity, and many of the behavioral responses associated with mania and drug addiction. Interestingly, a recent study by Yujnovsky et al. found that CLOCK:BMAL1 transcriptional activity is enhanced following dopamine D2 receptor stimulation in cell culture[70]. This increase in activity was mediated by an increased binding of the transcriptional coactivator, c-AMP-responsive element-binding protein (CREB) binding protein (CBP) to the CLOCK:BMAL1 complex. It is possible that activation of the D2 autoreceptors on dopaminergic neurons leads to an increase in the activity of CLOCK:BMAL1, thus changing the expression of genes like *TH* which results in a reduction in dopaminergic transmission.

It has not yet been determined whether restoration of CLOCK function in the VTA of the *Clock* mutant mice is sufficient to return their preference for cocaine to normal levels, and this will be investigated in the future. Likewise, the importance of proper CLOCK function in the SCN (or other brain regions) in these behavioral responses has not yet been investigated. In preliminary studies, we have found that mice with a mutation in *Npas2* have opposing responses in measures of drug reward to those seen in the *Clock* mutant mice (unpublished observations). This is very interesting since NPAS2 is very similar in structure and function to CLOCK, but has limited expression in the brain, including very little

**TABLE 1**
**A Comparison between the Behavioral Responses of the *Clock* Mutant Mice and the Common Symptoms of Mania***

| Symptoms of Mania | *Clock* Mutant Mice |
|---|---|
| Disrupted circadian rhythms | Disrupted circadian rhythms |
| Hyperactivity | Hyperactivity |
| Decreased sleep | Decreased sleep |
| Feelings of extreme euphoria | Hyperhedonia/less helplessness |
| Increased risk taking | Reduced anxiety |
| Propensity towards drug abuse | Increased preference for cocaine |

* Originally published in Roybal et al.[67].

(if any) expression in the SCN or the VTA[29]. Therefore, CLOCK and NPAS2 may perform different functions in the brain leading to differential regulation of drug reward.

## CONCLUSIONS

In conclusion, it seems likely that circadian rhythms and the genes that regulate the clock are involved in the responses to drugs of abuse and the vulnerability for addiction. Many studies have found diurnal differences in the sensitivity, sensitization, and reward value for drugs of abuse. There is also an increasing number of studies that find that circadian gene expression is regulated in response to drug treatment, and that animals lacking certain circadian genes display differences in the behavioral response to a variety of drugs. In addition, more investigators are beginning to look for variations in circadian gene sequences in people with various addictive disorders and, indeed, recent human genetic studies have even identified a polymorphism in the *Per2* gene that associates with heavy drinking among alcoholics[61]. It is becoming clear that the influence of the circadian genes on behaviors associated with addiction may involve the modulation of dopaminergic transmission between the VTA and NAc. This regulation might involve the circadian hormone melatonin, or the regulation of gene expression by the CLOCK protein and others in these dopaminergic neurons themselves. There is likely a strong postsynaptic component in striatal regions as well, since drugs of abuse reliably alter the expression of circadian genes in these regions. It is possible that the circadian genes have completely independent functions in these regions that are unrelated to their role in circadian rhythms. Therefore, the disruptions in sleep/wake and other rhythms in addicts would be secondary to disruptions in the proper function of these genes in regions of the brain that are important in drug responsiveness. It is also possible that these proteins form functional “clocks” in many brain regions (including the VTA and striatal regions) that become desynchronized form the SCN following drug treatment, and that this contributes to the development of addiction. Future work in this area will help to answer these questions and will undoubtedly lend new insight into the mechanisms that underlie the development of addiction.

## ACKNOWLEDGMENTS

I would like to thank Shibani Mukherjee, Edgardo Falcon, and Joseph Peevey for helpful comments on the manuscript. Work from our group was funded by NIDA, NIMH, and NARSAD.
